# Associations between food addiction symptoms, food intake and BMI-for-age in children from a low-income region: A structural equation modeling approach

**DOI:** 10.1038/s41430-026-01715-4

**Published:** 2026-03-03

**Authors:** Gabriela Carvalho Jurema Santos, Carol Góis Leandro, Tafnes Laís Pereira Santos de Almeida Oliveira, Isabella da Costa Ribeiro Nogueira, Ravi Marinho dos Santos, Jonathan Manoel da Costa, Rayssa Franciely Temudo dos Santos, Patrícia Miller Simões, Isabele Goés Nobre, Raquel Canuto

**Affiliations:** 1 Universidade Federal de Pernambuco (UFPE), Postgraduate Program in Nutrition, Recife, Pernambuco Brazil; 2 Universidade Federal de Pernambuco (UFPE), Postgraduate Program in Nutrition, Physical Activity and Phenotypic Plasticity, Centro Academico de Vitoria, Vitória de Santo Antão, Pernambuco Brazil; 3 Universidade Federal de Pernambuco (UFPE), Multicentric Postgraduate Program in Physiological Sciences, Vitória de Santo Antão, Pernambuco Brazil; 4 Universidade Federal do Rio Grande do Sul (UFRGS), Postgraduate Program in Nutrition. Department of Nutrition., Porto Alegre, Rio Grande do Sul Brazil

**Keywords:** Risk factors, Diseases, Epidemiology

## Abstract

**Background/objectives:**

Food addiction (FA) is characterized by the presence of dependence symptoms associated with the consumption of calorie-rich, sugary, and fatty foods, typical of ultra-processed foods. The growing prevalence of childhood obesity has been linked to the increased consumption of ultra-processed foods. This study aimed to analyze the direct and indirect associations between FA symptoms, food consumption, and body mass index for age (BMI-for-age) in children from 7 to 10 years of age from a Brazilian low-income region.

**Subjects/methods:**

A cross-sectional study was conducted, including 259 children of both genders, enrolled in public schools in the municipality of Vitória de Santo Antão, Pernambuco, Brazil. The Yale Food Addiction Scale for Children (YFAS-C) was used to assess FA symptoms. A food frequency questionnaire assessed food consumption, and the BMI-for-age was used to evaluate nutritional status. Structural equation analysis was employed for data analysis. The measurement model incorporated the seven FA symptoms from the YFAS-C, yielding favorable fit indices.

**Results:**

FA symptoms had an inverse direct effect on the consumption of *in natura* and minimally processed foods (β = -10.878; SE = 4.919; *p* = 0.027), while exhibiting a positive direct impact on the consumption of ultra-processed foods (β = 10.025; SE = 4.898; *p* = 0.001). The relationship between FA symptoms and BMI-for-age was not mediated by the consumption of ultra-processed foods (β = -0.054; SE = 0.081; *p* = 0.041).

**Conclusion:**

FA symptoms are associated with an increase in ultra-processed food consumption and a decrease in healthy food consumption among children from low-income families.

## Introduction

Excessive consumption of ultra-processed foods (UPF) is an increasingly prevalent problem among children [1]. The UPF consumption in childhood ranges from 15.9% (Colombia) to 57.5% (United States) of daily energy intake [2]. In Brazil, the consumption of UPF by children and adolescents corresponds to 25% of the total diet [3]. UPFs are industrial formulations characterized by excessive amounts of calories, sugar, and saturated fat, as well as the inclusion of additives [4].

The term UPF comes from the NOVA classification, which also categorizes foods into the following groups: *in natura* and minimally processed foods, which are obtained directly from plants or animals and consumed without any alteration after leaving nature, or that have undergone minimal processing to increase shelf life; culinary ingredients, which are substances extracted from natural foods through processes such as refining; and processed foods, which are natural or minimally processed foods with the addition of salt, sugar, oil, or other culinary ingredients to increase shelf life or enhance flavor [5].

High intake of UPF has been associated with excess weight and its indicators, such as body mass index for age (BMI-for-age) [6, 7]. Furthermore, excessive exposure in childhood may be a risk factor for adulthood obesity and changes in eating behavior. Childhood is a critical period of human development, and the first experiences are crucial for shaping eating habits [8]. In addition, schools in low-income areas provide a relevant setting for evaluation, due to greater exposure to UPF and present higher risks of nutritional inadequacies, including obesity [9].

UPF seem to have addictive potential due to their highly palatable composition, which is characterized by elevated levels of sugars, fats, salt, and flavor enhancers [10]. In adults, studies have well described the relationship between UPF and food addiction (FA) [11]; however, few studies have been conducted among children [12]. FA is characterized by the presence of dependence symptoms related to the consumption of palatable foods [13].

Its criteria follow those established for substance dependence in the Diagnostic and Statistical Manual of Mental Disorders - 5th edition (DSM-5), such as impaired control, social impairment, tolerance, and abstinence [14]. The Yale FA scale (YFAS) has been adopted for its identification, and it has a child population version (YFAS-C). The 25-item YFAS-C corresponds to seven symptoms that make up food dependence, such as persistent desire and continued use despite the consequences [15], and presents clearer and easier-to-understand language for children [16].

The relationship between FA and overweight seems to be partially mediated by UPF. However, no study has investigated the mediating role of food consumption in this relationship in children. This study aimed to analyze the direct and indirect associations of symptoms of FA, food consumption according to the NOVA classification, and BMI-for-age in children from 7 to 10 years of age from a Brazilian low-income region.

The study’s hypotheses (H) were: (H1) FA symptoms have a negative direct effect on the consumption of *in natura* and minimally processed foods; (H2) FA symptoms have a direct positive effect on the consumption of culinary ingredients, processed foods, and UPF; (H3) Consumption of culinary ingredients, processed foods, and UPF has a direct effect on BMI-for-age; (H4) Consumption of *in natura a*nd minimally processed foods has a negative direct effect on BMI-for-age; (H5) FA symptoms have a positive indirect effect on BMI-for-age mediated by the dietary consumption of UPF.

## Material and methods

A cross-sectional study was conducted from March 2022 to July 2023. Children of both sexes, aged 7 to 10 years, enrolled in public schools in the municipality of Vitória de Santo Antão, Pernambuco, Brazil, participated in the study. Vitória de Santo Antão is a city located 46 km from the capital, in the Northeast region, marked by the lowest rates of average household income per capita and Human Development Index. It has a population of 134,084 inhabitants and a municipal human development index below the national average (0.640 vs. 0.766 national average). It has a poverty rate of 49.8% and a Gini index of 0.42, reflecting social inequality and deprivation in access to education, healthcare, and financial resources [17].

The city has 24 schools, of which only three participated in the study due to availability. Consequently, 1150 children were invited to participate, and 273 agreed. Children who had difficulty responding to the questionnaires were excluded (*n* = 14) (cognitive limitations). A total of 259 children were included in the study analyses. Kline’s recommendation stipulated a minimum of 200 cases for conducting Structural Equation Modeling analysis [18].

### Food addiction symptoms: exposure variable

FA symptoms were investigated using the YFAS-C [15]. This questionnaire, adapted for children aged 4 to 16 years, was developed based on seven specific criteria resembling the symptoms of substance dependence as indicated in the Diagnostic and Statistical Manual of Mental Disorders – 4^th^ edition (DSM-IV) [19]. The translated and validated version for the Brazilian population was used [20].

The YFAS-C contains 25 items divided into a 5-point Likert scale with scores ranging from 0 (never) to 4 (always) (18 items) and dichotomous (yes/no) (5 items) questions that reflect seven symptoms related to addictive-like eating behavior: Symptom 1 - Substance taken in larger amounts and for a longer period than intended (items 1, 2, and 3); Symptom 2 - Persistent desire or repeated unsuccessful attempts to quit (items 4, 17, 18, and 25); Symptom 3 - Much time/activity to obtain, use, or recover (items 5, 6, and 7); Symptom 4 - Important social, occupational, or recreational activities given up or reduced (items 8, 9, 10, and 11); Symptom 5 - Use continues despite knowledge of adverse consequences (item 21); Symptom 6 - Tolerance (items 22 and 23); Symptom 7 - Substance taken to relieve withdrawal (items 12, 13, and 14). Although the scale consists of 25 items, only 22 are used to identify symptoms and included in the analyses, as items 19, 20, and 24 are not required for calculating the final score [15]. The YFAS-C was answered individually by the children with the help of an evaluator.

The present study focused on the assessment of symptoms, rather than diagnosis FA. The classification of each item for diagnosing symptoms followed the criteria from the development study [15]. After converting the scores into dichotomous values, the presence or absence of each symptom was assessed (yes/no) for descriptive analyses. Additionally, for the multivariable analyses, one latent continuous variable was derived through confirmatory factor analysis (CFA) [15].

### Food intake: mediator variables

Food consumption was assessed using an 81-item food frequency questionnaire (FFQ) that had been previously validated [21]. For each item, the frequency of consumption in the last month and portion size were obtained using a photographic album [22]. The food classification process was carried out in three stages using a decision tree based on the NOVA classification [23], which was used throughout the classification process.

In the first stage, three researchers independently pre-classified the foods in the FFQ according to their degree of processing. In the second stage, the researchers’ categorizations were triangulated. Foods for which there was consensus among all researchers were classified into their respective groups according to the NOVA food classification system. In cases of disagreement, a group discussion was held to reach a consensus, and the list of ingredients was considered, based on the NOVA classification. In the third stage, a group of experts, composed of two researchers with experience in evaluating UPF consumption, independently reviewed and discussed the categorization of pre-selected products. The evaluators involved in this stage were different from those in the previous stages.

At the end of this process, all foods were divided into one of four groups according to the NOVA classification [5]: (G1) *in natura* and minimally processed; (G2) culinary ingredients; (G3) processed; (G4) UPF **(**Supplementary Table 1). The daily amount consumed of each food consumed, in grams was, calculated by multiplying the frequency by the portion size. Next, we estimated the nutritional composition and energy consumption. The mean contribution (%) of each NOVA group to total energy intake was calculated.

### BMI-for-age Z index: outcome

Nutritional status was measured using the BMI-for-age Z index (BMI-for-age). Body weight was obtained using a platform scale with a maximum capacity of 150 kg and a precision of 100 g (Omron, HBF-214, São Paulo, Brazil). The participant was positioned standing, with their back to the scale’s height rod, in an upright posture and lightly clothed (wearing shorts and a t-shirt). Height was measured from the ground reference plane to the apex, with the individual barefoot, using a digital ultrasonic stadiometer (Avanutri, AVA-040, Rio de Janeiro, Brazil) with a measurement scale accurate to 0.1 cm [24]. BMI-for-age was then calculated using WHO AnthroPlus software (World Health Organization, Geneva, Switzerland, version 1.0.3). BMI-for-age was presented as both continuous and categorical data. For the categorical assessment, children were classified into two groups: normal weight (z score – 2 and +1) and overweight/obesity (z score ≥ +1) according to age and sex [25]. No participant was underweight.

### Covariates

Demographic variables included age (years), sex, and race/skin color (self-reported according to the categories of the Brazilian Institute of Geography and Statistics - IBGE: white, black, brown, yellow, and indigenous). Socioeconomic variables were evaluated using the Brazilian Economic Classification Criteria, which estimate the purchasing power of urban individuals and families. The instrument collects information on the possession and quantity of durable goods in the household, the number of bathrooms, the presence of a monthly employee in the household, and the level of education of the household head. Each item has a score, which is summed to classify families into categories A, B1, B2, C1, C2, D, and E [26]. Missing data on socioeconomic status resulted from non-responses by some participants.

### Statistical analyses

Data were entered into the Epidata program^®^ (version 3.1, Aarhus, Denmark). Statistical analyses were performed using STATA software^®^ (version 16.0, StataCorp, Texas, USA). Categorical data were presented in absolute and relative frequencies, while continuous variables were expressed as means and standard deviations, or medians and minimum and maximum values. Data normality was assessed using the Kolmogorov-Smirnov test. Differences between groups were evaluated using the Chi-square, Student’s t-test, and Mann-Whitney test. The intraclass correlation coefficient (ICC) was employed to evaluate interrater agreement in the classification of foods based on the NOVA classification system.

Structural equation modeling was employed to examine the relationships between symptoms of FA, food intake, and BMI-for-age. This was achieved through simultaneous CFA and regression analysis, with the model consisting of two parts: a measurement model and a path model.

The measurement model assessed the psychometric properties of the YFAS-C through CFA. A latent variable (L1) was derived from the seven FA symptoms (observed variables). This one-factor model aligns with the purpose of the validation study of YFAS [15].

The path models were developed to test the study hypotheses, which are based on the theoretical construct of the relationship between FA symptoms, food consumption, and BMI-for-age [27]. The models included the assessment of the association between FA symptoms and food intake, classified by the degree of food processing (G1, G2, G3, and G4) (direct effect), based on previously reported associations between FA symptoms and food intake [12]. In addition, it examined the association of the FA symptoms on BMI-for-age considering food intake as a mediating variable, according to the possible role of food intake on the relationship between FA and nutritional status [27]. Four path models were constructed, one for each food pattern group. No covariates were included in the models because there were no significant associations with BMI-for-age, dietary intake, and FA symptoms in the bivariate analysis (*p* < 0.05).

The models were estimated using maximum likelihood. Items with moderate factor loadings (≥ 0.3) were retained in the model. Model fit was assessed using the Chi-square test (*p*-value > 0.05), Comparative Fit Index (CFI > 0.95), Tucker-Lewis Index (TLI > 0.95), Root Mean Square Error of Approximation (RMSEA < 0.05), and Standardized Root Mean Squared Residual (SRMR < 0.08) [28] for both measurement and path models. Post-estimation commands were used to enhance the model’s fit by incorporating theoretically sound correlations among error terms. Standardized coefficients are presented in the results.

## Results

In total, 259 children of both sexes (50.6% girls), aged 7 to 10 years old, participated in the study. Of these, 74.5% identified themselves as brown, black, yellow, or indigenous, and 78% were from social classes C, D, or E. Regarding nutritional status, 63.3% of participants were eutrophic. G1 (50.3 ± 12.1) and G4 (38.1 ± 12.4) were the food groups with the greatest energy contributions to the diet (Table 1).

**Table 1:** Characteristics of study participants and energy intake (*n* = 259).

Age (years), % (*N*)	
7	27.4 (71)
8	21.2 (55)
9	26.3 (68)
10	25.1 (65)
Sex, % (N)	
Girl	50.6 (131)
Boys	49.4 (128)
Race, % (N)	
White	25.5 (66)
Brown/Black/Yellow/Indigenous	74.5 (193)
Socioeconomic level, % (N)*	
A/B1/B2	7.7 (20)
C1/C2/D/E	78.0 (202)
BMI-for-age, median (min-max)	0.39 (-3.4 – 5.3)
Nutritional status, % (N)	
Eutrophic (< Z + 1)	63.3 (164)
Overweight/obesity (≥ Z + 1)	36.7 (95)
Energy contribution (% total calories ingested per day), mean ± DP	
G1	50.3 ± 12.1
G2	1.72 ± 2.5
G3	9.8 ± 5.6
G4	38.1 ± 12.4

*BMI-for-age* Body mass index for age, *G*, *in natura* and minimally processed, *G2* culinary ingredients, *G3* processed, *G4* ultra-processed. *Missing: 14.3% (*n* = 37).

The description of sociodemographic variables and nutritional status according to FA symptoms can be observed in Table 2. No differences were observed between FA symptoms and sex, race, and nutritional status. All FA symptoms were significantly associated with the frequency of FA, and age and socioeconomic level were associated with symptoms 2, 4, and 6. No associations were observed between nutritional status and socioeconomic and demographic variables (data not shown in tables).

**Table 2:** Symptoms of food addiction according to socioeconomic characteristics and nutritional status.

	Symptoms																
	1	p	2		p	3	p	4	p	5	p	6			p	7	p
Frequency, % (N)	30.9 (80)	<0.001	52.5 (136)		0.022	25.1 (65)	<0.001	52.1 (135)	0.019	25.1 (65)	<0.001	52.5 (136)			0.022	25.1 (65)	<0.001
Age (years), % (N)		0.408			0.023		0.624		0.037		0.624				0.023		0.624
7	21.3 (17)		23.5 (32)			33.8 (22)		23.7 (32)		33.8 (22)		23.5 (32)				33.8 (22)	
8	25.0 (20)		24.3 (33)			20.0 (13)		24.4 (33)		20.0 (13)		24.3 (33)				20.0 (13)	
9	31.3 (25)		20.6 (28)			23.1 (15)		20.7 (28)		23.1 (15)		20.6 (28)				23.1 (15)	
10	22.5 (18)		31.6 (43)			23.1 (15)		31.1 (42)		23.1 (15)		31.6 (43)				23.1 (15)	
Sex, % (N)		0.869			0.526		0.199		0.449		0.199				0.377		0.199
Girls	50.0 (40)		48.5 (66)			43.1 (28)		53.2 (66)		43.1 (28)		47.8 (65)				43.1 (28)	
Boys	50.0 (40)		51.5 (70)			56.9 (58)		46.8 (58)		56.9 (37)		52.2 (71)				56.9 (37)	
Race, % (N)		0.129			0.738		0.249		0.760		0.249				0.835		0.249
White	26.3 (21)		26.8 (33)			22.7 (15)		24.3 (33)		22.7 (15)		24.8 (34)				22.7 (15)	
Brown/Black/Yellow/Indigenous	72.6 (58)		70.7 (87)			77.4 (51)		75.3 (103)		77.3 (51)		75.1 (103)				77.7 (51)	
Socioeconomic level, % (N)^#^		0.281			0.004		0.807		0.005		0.807				0.004		0.807
A/B1/B2	3.8 (3)		5.1 (7)			7.7 (5)		5.2 (7)		7.6 (5)			5.1 (7)			7.6 (5)	
C1/C2/D/E	85.0 (68)		86.8 (118)			81.5 (53)		86.7 (117)		81.8 (54)			86.9 (119)			81.8 (54)	
BMI-for-age, median (min-max)	0.40 (3.4 – 5.3)	0.433	0.42 (-2.0 – 4.7)	0.732		0.21 (-2.0 – 3.6)	0.280	0.42 (-2.0 – 4.7)	0.663	0.21 (-2.0 – 3.6)	0.280		0.41 (-2.0 – 4.7)	0.732		0.21 (-2.0 – 3.6)	0.280
Nutritional status, % (N)		0.494		0.874			0.468		0.874		0.468			0.902			0.468
Eutrophic (< Z + 1)	60.2 (48)		62.8 (86)			69.7 (46)		62.5 (85)		69.7 (46)			62.8 (86)			69.7 (46)	
Overweight/obesity (≥ Z + 1)	38.8 (31)		36.5 (50)			30.3 (20)		36.8 (50)		30.3 (20)			36.5 (50)			30.3 (20)	

Symptom 1, Ingestion of the substance in greater quantity and for a longer period than intended; Symptom 2, Persistent desire or repeated unsuccessful attempts to give up; Symptom 3, Too much time spent obtaining and using substance or recovering from its effects; Symptom 4, Abandonment and reduction in carrying out social, occupational or recreational activities due to substance use; Symptom 5, Continued use of the substance despite knowledge of the adverse effects; Symptom 6, Tolerance; Symptom 7, withdrawal. A/B1/B2, higher economic classes; C1/C2/D/E, lower economic classes; *N* number of participants; %, frequency; p, *p* value; ^#^Missing: 14.3% (*n* = 37); Frequency comparison of qualitative variables performed using the chi-square test.

In the assessment of interrater agreement for the NOVA classification, an ICC of 0.98 was observed. The description according to the NOVA classification and FA symptoms is provided in Table 3. Higher consumption of UPF was observed in symptoms 1, 2, 4, and 6. On the other hand, higher consumption of *in natura* and minimally processed foods was observed in the absence of these symptoms.

**Table 3:** Food consumption according to degrees of food processing and FA symptoms.

	G1 (%kcal)		G2 (%kcal)		G3 (%kcal)		G4 (%kcal)	
		p		p		p		p
Symptom 1		0.028		0.893		0.415		0.005
Yes	47.8 ± 11.2		0.8 (0.0 – 18.0)		9.3 ± 5.0		40.6 (2.1 – 70.7)	
No	51.4 ± 12.4		0.7 (0.0 – 16.0)		10.0 ± 5.8		36.6 (3.0 – 72.1)	
Symptom 2		0.012		0.804		0.960		0.014
Yes	48.5 ± 13.0		0.7 (0.0 – 18.0)		9.8 ± 6.0		39.8 ± 13.6	
No	52.3 ± 10.8		0.7 (0.0 – 16.0)		9.8 ± 5.0		36.1 ± 10.6	
Symptom 3		0.739		0.476		0.803		0.845
Yes	50.8 ± 12.6		0.6 (0.0 – 18.0)		9.6 ± 4.6		37.7 ± 13.6	
No	50.2 ± 12.0		0.8 (0.0 – 16.0)		9.8 ± 5.9		38.2 ± 12.0	
Symptom 4		0.004		0.901		0.935		0.020
Yes	48.4 (18.8 – 83.2)		0.7 (0.0 – 18.0)		9.8 ± 6.0		39.7 ± 13.6	
No	52.3 (26.3 – 87.5)		0.7 (0.0 – 16.0)		9.7 ± 5.0		36.2 ± 10.6	
Symptom 5		0.739		0.476		0.803		0.845
Yes	50.8 ± 12.6		0.6 (0.0 – 18.0)		9.6 ± 4.6		37.7 ± 13.6	
No	50.2 ± 12.0		0.8 (0.0 – 16.0)		9.8 ± 5.9		38.2 ± 12.0	
Symptom 6		0.006		1.00		0.948		0.021
Yes	48.6 (18.8 – 83.2)		0.7 (0.0 – 18.0)		9.7 ± 6.0		39.7 ± 13.6	
No	52.2 (26.3 – 87.5)		0.7 (0.0 – 16.0)		9.8 ± 5.0		36.2 ± 10.7	
Symptom 7		0.739		0.476		0.803		0.845
Yes	50.8 ± 12.6		0.6 (0.0 – 18.0)		9.6 ± 4.6		37.7 ± 13.6	
No	50.2 ± 12.0		0.8 (0.0 – 16.0)		9.8 ± 5.9		38.2 ± 12.0	

G1, *in natura* and minimally processed; G2, culinary ingredients; G3, processed; G4, ultra-processed. Symptom 1, Ingestion of the substance in greater quantity and for a longer period than intended; Symptom 2, Persistent desire or repeated unsuccessful attempts to give up; Symptom 3, Too much time spent obtaining and using substance or recovering from its effects; Symptom 4, Abandonment and reduction in carrying out social, occupational or recreational activities due to substance use; Symptom 5, Continued use of the substance despite knowledge of the adverse effects; Symptom 6, Tolerance; Symptom 7, withdrawal.Test t-student and Mann-Whitney. Parametric data are presented as mean ± standard deviation and non-parametric as median minimum and maximum values.

The fit indices of the measurement models are presented in Table 4. All indices were considered adequate. We began the analysis by confirming the factor structure of the seven symptoms of YFAS-C, and good adjustment indices were observed.

**Table 4:** Model fit to evaluate the association of direct, indirect, and total effects between FA symptoms, degree of food processing and BMI-for-age.

Model	X^2^	RMSEA	RMSEA 90% CI	SRMR	CFI	TLI
FA symptoms	18.069 (*p* = 0.204)	0.034	0.000 – 0.074	0.041	0.966	0.949
G1	48.763 (*p* = 0.220)	0.031	0.000 – 0.061	0.048	0.951	0.935
G2	29.045 (*p* = 0.359)	0.018	0.000 – 0.054	0.043	0.983	0.977
G3	24.254 (*p* = 0.616)	0.000	0.000 – 0.043	0.039	1.000	1.030
G4	35.226 (*p* = 0.133)	0.035	0.000 – 0.064	0.049	0.936	0.915

*G1* *in natura* and minimally processed, *G2* culinary ingredients, *G3* processed foods, *G4* ultra-processed foods; X^**2**^ chi-square test; *RMSEA* Root Mean Square Error of Approximation fit index, *SRMR* Standardized Root Mean Square residual, *CFI* comparative fit index, *TLI* Tucker-Lewis’s index.

**Fig. 1:**
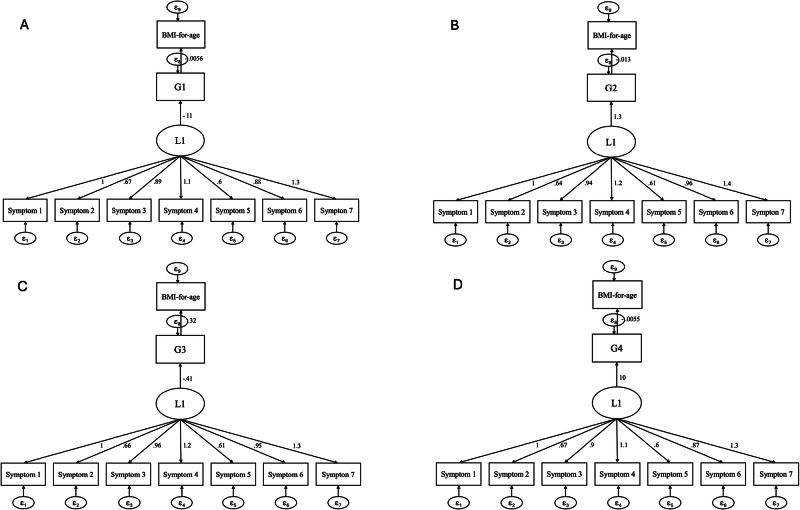
Structural equation modeling of FA symptoms (L1), food consumption according to the degree of food processing and BMI-for-age. Caption: [G1 = *in natura* and minimally processed (**A**), G2= culinary ingredients (**B**), G3= processed foods, (**C**) and G4= ultra-processed foods (**D**). All estimates are standardized.

The four models of direct and indirect associations between FA symptoms, food intake, and BMI-for-age are presented in Fig. 1 and Table 5. Confirming hypothesis H1, FA symptoms had a negative direct effect on the consumption of *in natura* and minimally processed foods (β = -10.878; SE = 4.919). No direct effects were observed between FA symptoms and culinary ingredients (β = 1.321; SE = 0.969) and processed foods (β = -0.409; SE = 2.233). However, a positive direct effect of FA symptoms on the consumption of UPF was observed (β = 10.025; SE = 4.898), partially confirming H2 (Table 5 and Fig. 1).

**Table 5:** Standardized total effect, direct effect, and indirect effect in the Structural Equation Model of the latent variable of FA symptoms, food intake and BMI-for-age.

FA symptoms	BMI-for-age		
*In natura* and minimally processed (G1)	β	SE	*P* value
*Direct effect*			
FA symptoms → G1	**-10.878**	**4.919**	**0.027**
G1 → BMI-for-age	-0.005	0.007	0.469
*Indirect effect*			
FA symptoms → G1 → BMI-for-age	0.060	0.088	0.491
*Total effect*			
FA symptoms → G1 → BMI-for-age	0.060	0.088	0.491
**Culinary ingredient (G2)**			
*Direct effect*			
FA symptoms → G2	1.321	0.969	0.173
G2 → BMI-for-age	-0.013	0.039	0.738
*Indirect effect*			
FA symptoms → G2 → BMI-for-age	-0.017	0.053	0.745
*Total effect*			
FA symptoms → G2 → BMI-for-age	-0.017	0.053	0.745
**Processed foods (G3)**			
*Direct effect*			
FA symptoms → G3	-0.409	2.233	0.854
G3 → BMI-for-age	**0.054**	**0.016**	**0.001**
*Indirect effect*			
FA symptoms → G3 → BMI-for-age	-0.022	0.122	0.855
*Total effect*			
FA symptoms → G3 → BMI-for-age	-0.022	0.122	0.855
**Ultra-processed foods (G4)**			
*Direct effect*			
FA symptoms → G4	**10.025**	**4.898**	**0.041**
G4 → BMI-for-age	-0.005	0.007	0.474
*Indirect effect*			
FA symptoms → G4 → BMI-for-age	-0.054	0.081	0.499
*Total effect*			
FA symptoms → G4 → BMI-for-age	-0.054	0.081	0.499

*G1* *in natura* and minimally processed, *G2* culinary ingredients, *G3* processed foods, *G4* ultra-processed foods, *BMI* body mass index, *FA* food addiction. Values in bold indicate statistical significance.

When evaluating the association between the degree of food processing and BMI-for-age, a positive direct effect was observed only between the consumption of processed foods and BMI-for-age (H3) (β = 0.054; SE = 0.016). No effects were observed in the other food groups, including *in natura* and minimally processed foods that were not directly associated with a negative effect on BMI-for-age (H4) (β = 0.0005; SE = 0.0007). Additionally, the relationship between FA symptoms and BMI-for-age is not mediated by the consumption of UPF, refuting our hypothesis H5 (β = -0.054; SE = 0.081) (Table 5 and Fig. 1).

## Discussion

This is the first study to demonstrate that children with fewer FA symptoms have a greater consumption of *in natura* and minimally processed foods, supporting our hypothesis H1. Furthermore, we demonstrate that children with increased FA symptoms consume more UPF (H2). However, food consumption did not mediate the relationship between FA symptoms and BMI-for-age (H5).

The consumption of UPF has been associated with FA and its symptoms in previous studies [29–31]. FA symptoms are considered a predictive factor for UPF intake [32]. In children (9 to 11 years), abstinence and continued use of the substance were associated with daily energy consumption and sugar intake, respectively [29]. However, in childhood, these relationships are still little explored. A systematic review identified that FA in children and adolescents is associated with consumption of calories, carbohydrates, proteins, fats, sodium, sugar, and UPF [12].

The factors that describe the association between FA and UPF are still unknown. The greater preference for UPF in individuals with FA has been associated with reduced activation in brain regions linked to the reward system and a decrease in inhibitory control [30]. These responses can be maintained and reinforced by chronic compulsive consumption of these foods [33].

On the other hand, natural foods promote low activation of reward pathways due to their nutritional composition, which do not have properties that favor addiction [15]. This occurs because they do not contain high concentrations of sugar and fat simultaneously and are not absorbed quickly, excessively increasing blood sugar levels [34].

Income-related factors have impacted UPF consumption. Social inequality, access to advertising, and reduced food costs are associated with UPF consumption [35]. These results have been described even in children’s populations. For example, Nepalese children have a high daily consumption of UPF ( > 60%), such as sugary drinks and processed snacks [36]. In India, more than 80% of children (3-6 years old) assessed had been introduced to daily junk food early in life and lived within a 5-min walk of a junk food store [37]. Furthermore, adolescents (age 15.03 ± 0.16) from rural area of Bangladesh were less likely to consume sugary drinks when they had higher education [38].

A trend observed is the reduction in the consumption of *in natura* and minimally processed foods in childhood, especially in low-income populations, due to low availability [39]. In children, increased consumption of *in natura* and minimally processed foods was associated with reduced BMI-for-age, waist circumference, serum insulin, and blood pressure [40]. On the other hand, changes in parameters related to nutritional status have been observed when there is an increase in the consumption of processed and UPF [1, 41].

Although previous studies conducted in similar populations have reported associations between sociodemographic factors and nutritional status, our study did not observe any such associations. A study conducted in 2019 with 6288 Nepalese children (under five) reported an association between height-for-age, an indicator of nutritional status, and factors such as age, sex, province, and household wealth [42]. Similarly, another study involving 21,477 children under five in the Democratic Republic of the Congo found that family income and maternal education were significantly associated with children’s nutritional status [43]. This discrepancy may be attributed to the limited variability in sociodemographic characteristics within our sample (with 78% of participants belonging to social classes C/D/E).

In our study, it was possible to observe an association between processed foods and BMI-for-age. Similar outcomes have been observed, where the consumption of processed foods was positively associated with BMI-for-age and overweight [44, 45]. Processed foods are typically characterized by the addition of salt, sugar, and fats. Guidelines and government programs have recommended moderate consumption of processed foods and a reduction of UPF, given their relationship with changes in body composition [46].

On the other hand, UPF was not associated with BMI-for-age, possibly due to the limited variability in intake among participants. These foods were consumed in substantial amounts across all BMI-for-age categories, which may have masked detectable differences. In the study by Costa et al. (2023), higher UPF scores were associated with higher BMI-for-age [47]. In this context, the absence of percentile-based assessment in the present study may have limited the detection of associations between UPF intake and BMI-for-age.

It was also observed that dietary intake was not a mediating factor between FA symptoms and BMI-for-age. Although the literature demonstrates an association between these parameters, we did not observe this relationship, probably due to the limitations of the BMI-for-age indicator in demonstrating variations in body composition and because it is a growth and development phase characterized by high metabolic and physiological variability [48, 49].

This article contributes to the understanding of FA symptoms and their consequences on health, especially in childhood. Studies on FA are relatively new, and its concept is still under development. However, its impacts and relationships are well-established in the literature [50].

A strong point of this study was the use of structural equation modeling as a technique for measuring relationships through linear regression, CFA, and path analysis. The difficulty in diagnosing FA in children, due to problems interpreting the YFAS-C, may reveal different frequencies of FA [12]. Therefore, using symptoms to create a latent variable may be an alternative to test hypothesized relationships based on FA symptoms [51].

Another strong point was the description of the effects of FA symptoms in the child population. Childhood corresponds to an age group where physical, cognitive, emotional, and social development occurs [52]. Evidence shows that nutritional deprivation during childhood can alter the structure and function of the brain, including the areas responsible for controlling appetite and reward. These changes can lead to a greater preference for foods high in calories and fat [53]. Furthermore, this can be exacerbated in situations of food insecurity [54].

Our study found some limitations. First, the use of the YFAS-C is based on DSM-IV. Due to the lack of availability of more up-to-date instruments with language adapted for the target audience, it was not possible to use the recent version (YFAS-C 2.0), developed for adolescents [55]. Second, the use of a convenience sample. Third, the use of FFQ may accompany memory bias. Strategies were used to minimize bias, such as the use of photographic album, the multiple-pass method, and team training [56].

It is concluded that FA symptoms are directly associated with the consumption of UPF and inversely with *in natura* and minimally processed foods. Furthermore, it was not possible to identify the mediating effect of food consumption between FA symptoms and BMI-for-age. Additionally, the importance of adopting regulatory measures aimed at reducing the consumption of UPF is reinforced, including product surcharges, front-of-package labeling, and regulating advertising to children.

## Supplementary information

Supplementary table 1

## Data Availability

The datasets generated during and/or analyzed during the current study are available from the corresponding author on reasonable request.
