# Supplementary material for: Associations between food addiction symptoms, food intake and BMI-for-age in children from a low-income region: A structural equation modeling approach
Source: Eur J Clin Nutr. 2026 Mar 3;80(5):515–23. doi: 10.1038/s41430-026-01715-4 (PMC13186694; doi:10.1038/s41430-026-01715-4)
Supplement: Supplementary file 1 — Supplementary table 1 [file 41430_2026_1715_MOESM1_ESM.docx]

| **Supplementary table 1.** Classification of food frequency questionnaire items according to food processing characteristics | |
| --- | --- |
| **NOVA classification** | **Foods** |
| G1: *in natura* and minimally processed | Rice, sweet potato, couscous, cassava flour, yam, spaghetti, cassava, homemade popcorn, *tapioca*, beans, pineapple, avocado, banana, guava, *jaca,* orange, apple, mango, watermelon, fruit salad, *siriguela*, grape, beef, pork, chicken, chicken giblets, egg, fish, bovine viscera, nuts, lettuce, onion, carrot, pumpkin, tomato, liquid whole milk, powdered milk, *munguzá, pirão*, mashed potatoes, soup, coconut water, coffee, fruit juice and vitamin. |
| G2: culinary ingredients | Sugar and butter |
| G3: processed foods | Homemade cake, bread, jerked beef, yellow cheese, curd cheese and *farofa*. |
| G4: ultra-processed | Cracker, papa, sausage, ham, steak, margarine, chocolate powder, liquid chocolate, cookie without filling, cookie with filling, industrialized cake, chocolate, lollipop, ice-cream, yogurt, creamy cheese, french fries, fried savory snacks, instant noodles, lasagna, industrialized popcorn, pizza, packet snack, hot dog, soft drink, boxed juice, and powdered juice. |
